# Has the time come to teach parallel programming to secondary school students?

**DOI:** 10.1016/j.heliyon.2021.e08662

**Published:** 2021-12-27

**Authors:** Predrag Brođanac, Josip Novak, Ivica Boljat

**Affiliations:** a Faculty of Science, University of Split, Zagreb, Croatia; b Faculty of Humanities and Social Sciences, University of Zagreb, Croatia

**Keywords:** Parallel programming, Secondary school, Student attitudes, Ability to communicate concepts

## Abstract

Today, almost every computer has at least one multicore processor. To remain in stride with hardware developments, numerous university faculties oriented towards computer science have introduced parallel programming as an integral part of their courses. The question is, given the availability of parallel architectures, and considering future trends in programming, whether it is time for parallel programming to also become an integral part of the informatics curriculum in secondary schools? This paper presents research conducted in three schools in Croatia over several school years. A total of 162 students from the science-mathematic high schools participated in the research. The results, based on student evaluations, suggest that this course content is equally interesting and somewhat more difficult, and perceived as equally useful as other course content taught to students. Moreover, the findings indicate that students can understand and later apply some of the fundamental concepts of parallel programming.

## Highlights

• Can secondary school students understand the basic concepts of parallel programming?
• An example of teaching parallel programming to secondary school students.
• Parallel programming in Python using threads and processes.

## 1 Introduction

Between 1986 and 2002, the processor speed had increased on average by 50% each year [1], but has subsequently been decreasing on average by 20% each year [2]. Over the years, we have become accustomed to successive increases in processor power and have begun to use computers in various spheres of life. Processor power is not essential for the everyday use of computers. However, today computers are used a lot in scientific research such as drug research, genome decoding, the analysis of large datasets, and various simulations such as, for instance, modeling in biochemistry, climatology, nuclear reactions, and earthquakes. In these situations, performance is an important factor [3, 4].

Since 2005, most processor manufacturers have decided to focus on producing multicore processors, i.e., multiprocessor computers [5]. Today, this is most often based on 4 or 8 cores, but the assumption is that this will reach 100 cores in subsequent years [6].

Multiprocessor and multicore computers have become widely used [7], while the question about what point we have reached as programming educators remains. Though parallel programming has advanced in terms of tools, modern-day programmers should possess the know-how and experience to create applications for multiple processors. When parallel programming appeared, it was limited to a small, specialized community with access to multiprocessor systems [8]. As is the case with most discoveries, once they appear, they eventually reach the university level. This was the case with object-oriented programming back in the 1970s, as well as parallel programming in the mid-1990s [9]. We have witnessed that some of these concepts eventually reach lower levels of education institutions, for instance, object-oriented programming is today part of the curriculum in some secondary school programs [10, 11]. One of the conclusions at the conference Revitalizing Computer Architecture Research, held in 2005, was that parallel programming should be available to future programmers [12]. A few years later (2013), ACM/IEEE and NSF/IEEE-TCP recommended that parallel and distributed programming should be introduced into every undergraduate program in informatics [13]. W. B. Gardner [14] concluded that, since parallel architecture is commonly used nowadays, students should be familiar with it and get practical experience. Most researchers believe that parallel programming should be taught earlier in education [15, 16, 17]. Often, parallel programming is implemented as part of introductory programming courses, the so-called CS1 and CS2 [18, 19]. A large number of universities have changed their curriculums or modified existing courses, positioning parallel programming early in studies [20]. In the case of teaching parallel programming before university level, the research appears to be scarce. A few researchers have described their experience with parallel programming in secondary schools [21, 22, 23, 24, 25, 26].

### 1.1 The researcher motivation

Since parallel architecture is widely used and software solutions for parallel programming have been significantly advanced, the following question logically follows – can parallel programming in some form be taught at the secondary school level? Based on the available studies, there were only a few attempts of teaching parallel programming to secondary school students. So far, some other concepts, such as object-oriented programming have been taught at the secondary school level. It is not certain whether secondary school students can master basic concepts of parallel programming, such as fork/join, face condition, parallel speedup, or load balance. In addition, the question of how to motivate students to learn the concepts and how to present them to students remains.

### 1.2 The main contribution

A way to motivate students for learning parallel programming in terms of two aspects is elaborated in the paper: a) learning network application programming, which students are familiar with from previous lessons, and b) using problems students have previously encountered. An example of how to introduce programming within 12 school hours is provided. Furthermore, the paper presents the results of research, which suggest science-mathematics high school students, who attend informatics for four years for 105 school hours yearly, can understand race condition, one of the basic parallel programming concepts, and apply it successfully to other problems. Also, it is demonstrated that students consider parallel programming equally interesting and useful, but slightly more difficult, compared to the rest of the informatics topics.

### 1.3 Paper outline

The paper is organized as follows: a brief introduction to parallel programming is provided, followed by an overview of previous research in this area. The overview includes the findings on when and how it is appropriate to teach parallel programming. In the following sections, the conducted research is described and its findings are discussed, including open issues for future research.

### 1.4 Method

The literature search was done using ACM database (https://dl.acm.org) using keywords “parallel programming education.” The search yielded 166 237 results ordered by relevance. The first few papers were:

• *Teaching parallel programming using Java,*
• *A breadth-first course in multicore and many core programming,*
• *A Three-Semester, Interdisciplinary Approach to Parallel Programming in a Liberal Arts University Setting,*
• *A Three-Semester, Interdisciplinary Approach to Parallel Programming in a Liberal Arts University Setting, etc.*

Only papers which included parallel programming in general or parallel programming education notions in the title were included, preferring papers that include writing parallel program scripts. Each of these papers was included if considered relevant based on the abstract. The included papers were downloaded and listed in the MS Excel table.

In total, 203 papers were identified. During the literature screening, brief annotations about the paper content were made. For instance, whether the content includes teaching parallel programming in secondary schools, college, or if it is a substantial research. After the screening, 115 papers were rejected for not being relevant to the study aim.

## 2 Experience in teaching parallel programming

There are many types of hardware architectures, as well as approaches to parallel programming in a single type of parallel architecture [33]. Accordingly, there are currently numerous models used for teaching parallel programming and it remains unknown which model will eventually prevail [6].

As is the case for teaching other programming concepts, the program environment is important but must not be the sole factor. The basic reason for this are frequent changes in program environments [34]. L. Ivanov [35] takes this a step further and suggests that parallel programming is one of the most dynamic areas of computer science. Carro et al, [36] believe that it is better to teach parallel programming conceptually than rely on a particular program environment.

Brown et al, [37] presented their views on 4 module levels for teaching parallel programming:

• Very low-level libraries closely tied to hardware and operating systems,
• Very low-level libraries with a layer for abstraction and management,
• Programming languages that support parallel programming,
• High-level environments for productive parallel programming.

Most of today's desktops have two or more cores, or otherwise two or more processors [7]. Today, multicore processors are also installed into tablets, smartphones, and similar devices [16]. We have also seen several program environments for parallel programming (threads, MPI, MapReduce, OpenMP) in various programming languages (Python, C/C++, Java, C#, Erlang, Scala). However, the situation was different 30 years ago. Parallel hardware infrastructure was expensive and difficult to get, and the situation with program environments for the development of parallel programs was hardly any better. In addition, the method of teaching parallel programming was not developed.

A. Radenski [38] described the possibility of parallel programming in cloud but underscores the lack of the appropriate hardware. He considered the parallel programming architecture expensive but not used for the whole time it was paid for, so it can be practical to rent a cloud computer with desired characteristics when required. Khorsand et al, [39] and Khorsand et al, [40] describe the system which suggests the optimal architecture in terms of cost/response cloud system.

Though perhaps at first glance it may seem that parallel programming hardly differs from standard (serial) programming, experience has shown that students have difficulties with parallel programming [41]. Authors cite some of the basic reasons for these problems:

• Parallel programming requires a different way of thinking,
• Parallel programs are dynamic, which makes debugging more difficult,
• Proper program synchronization is difficult to achieve.

Besides standard mistakes in parallel programming, which also occur in serial programming, there are other types of mistakes not occurring in serial programming, such as [42]:

• Data race – occurs when two or more threads simultaneously read or write to the same memory location,
• Deadlock – occurs when a thread is waiting for conditions which are never fulfilled,
• Miscellaneous use of variable locking.

A research conducted on 180 student solutions [42] found that 23 solutions include a data race mistake, where the variable protection mechanism was not used, followed by 11 solutions in which the program underwent a deadlock, while 11 students unnecessarily used variable locking in programs.

Numerous documents, among which are ACM CS2013 curriculum guidelines and NSF/IEEE-TCPP [43], testify that parallel programming should be introduced for all computer science students. ACM CS2013 recommends that parallel programming be dispersed across several courses throughout the studies. This leads to certain administrative challenges [44], some of which are:

• Determining which courses should include parallel programming – serial programming is a special case of parallel programming, hence parallel programming can be included in initial programming courses,
• Adaptation of course content – introducing new concepts means that some existing ones need to be eliminated,
• Education of teaching personnel.

In their research, Feldman and Bachus [17] showed that parallel programming can be introduced in earlier years of studies. Plouzeau and M. Raynal [45] describe what a parallel programming course should contain and consider that the fundamentals of the parallel programming courses include becoming familiar with serial programming.

Parallel programming within the framework of CS1 and CS2 courses is described by D. Valentine [18], who notes that 100% of students consider that informatics students should know the basics of parallel programming after completing the third year of their studies. Experience in integrating fundamentals of parallel programming into two existing courses within an undergraduate program was addressed by Bunde et al, [46]. It totaled 2.5 h (1.5 h of lectures and 1 h of computer exercises) in the first course, where students became familiar with data exchange between the CPU and GPU, and which is supported by an example for summing two vectors, while the topic in the second course was a parallel game of life.

D. G. Hyde [33] suggests that parallel programming should be a separate course in the undergraduate program. He also notes that parallel programming is natural, given that the world which surrounds us is parallel. The reason behind the difficulties in understanding parallel programming lies perhaps in choosing the wrong programming language. Moreover, the author believes that parallel programming requires doing computer exercises and cites a series of problems suitable for studying parallel programming. Lu et al, [47] describe a course that can be taught possibly before all other introductory courses (CS0), i.e., an advanced placement subject for secondary school students, covering the fundamentals of working with computers and programming, and describing parallel programming topics. Interestingly, the course begins with the programming language Scratch, in which the authors advocate learning through experience.

Authors Torbert et al, [26] describe their enviable experience in teaching parallel programming as an elective subject in secondary school in the 1990s. The programming language they used is C, combined with the tool for parallel programming MPI. In the second part of the subject, XMT, pthreads, OpenMP, sockets, and programming of graphics processors and CUDA are used for parallel programming. Examples they use to teach parallel programming are related to searching, fractal generation, cellular automata, image processing, and matrix operations. Importantly, this subject is only for the most successful students in programming. Parallel programming as an elective subject in secondary school (final year) using the programming language Pascal was addressed by Ben-David Kolikant [21]. The goal of the undertaken research was to gain an understanding of synchronization a month after completing the subject. The results indicated that students had a good understanding of most of the synchronization-associated problems, but most of them drew conclusions based on a certain template. Another identified problem was the relationship between parallel programming and previously taught content. Studying parallel programming during a three-day camp for selected students was described by Chesebrough and Turner [48]. At the camp, in addition to role-playing and programming in C/C++ and OpenMP, standard parallel problems were also treated such as parallel processing of list elements, matrix multiplication, calculating the value of the number π. Interestingly, students expressed more positive attitudes towards programming in OpenMP compared to role-playing, which some of the students thought was childish. Similar experience in teaching parallel programming for children aged 9–10 possessing no programming experience was described by Gregg et al, [49]. The fundamental goals of the workshop were the adoption of programming fundamentals in multiprocessors, understanding programming and computers, and teaching students parallel thinking. The tool *EcoSim* was devised for developing parallel programming, which is similar to spoken language. Experience in teaching parallel programming in the programming language *Scratch* for girls in later years of primary and secondary school was described by Feldhausen et al, [22]. Over 80% of students in the camp believed they were able to apply their knowledge to some other parallel problems in *Scratch*, and that they understood parallel programming. A description of the workshop lasting up to 50 min for secondary school students, novice in programming, where the goal was to show that students can understand the concepts of parallel programming is given by A. Rifkin [23].

Broll et al, [50] believes that well-devised teaching materials can help students in K-12 master the fundamentals of parallel programming. To help students achieve that, the authors created *NetsBlox* – a program similar to *Scratch*, which has its advantages in creating parallel applications. *NetsBlox* can create games where parallelism is achieved in two ways: two characters are doing something at the same time or one of the characters is doing several tasks simultaneously (e.g., moving and also collecting some elements on a board). In general, learning programming through play can positively influence student motivation and result in improved test performance [51].

Robots have several sensors to collect various data which is simultaneously processed and result in a certain action. It is possible to use a system of heterogeneous robots to do chores for the elderly according to the task allocation [52]. A similar example in terms of parallel programming was presented by Bordignon et al, [53]. Jacobsen and Jadud [54] elaborate on introducing parallel programming using robots, and assume this will help students gain a deeper understanding of parallel programming, and make it more interesting.

M. Paprzycki [55] believes that parallel programming is natural, and that serial programming is only a particular instance of parallel programming. Therefore, the author believes that teaching programming should begin with parallel programming.

Some of the standard examples for teaching parallel programming are:

• Searching [26, 35, 48],
• Fractal generation [26, 35, 48],
• Cellular automata [26, 34, 35, 56],
• Matrix operations [33, 34, 48, 56, 57, 58],
• Computing the value of the number π [35, 48, 56],
• Sorting [23, 33, 34, 57, 59],
• Cracking passwords [60, 61],
• Adding elements to a list [22, 62],
• Prime numbers [59, 61, 63],
• Image processing [56, 64],
• Client-server applications [65].

## 3 Method

Numerous authors (e.g. [7, 12, 66, 67], and [57]) suggest that parallel programming should be introduced into the curriculum early, prior to familiarizing students with serial programming. D. H. Clements [68] asserts that transferring from serial to parallel programming is difficult and that parallel programming should take place earlier in the curriculum. M. Ben-Ari [69] consider that parallel programming should be taught earlier than is the current practice, even as early as secondary schools. A. Rifkin [23] believes that secondary school students should be learning serial and parallel programming simultaneously. An argument supporting this is that teaching serial programming is, to a large extent, an obstacle on the path to parallel thinking, where serial types of solutions are mostly sought for problems. Broll et al, [50] suggest that well-devised examples in parallel programming can be taught even before enrolling in a university program.

Experience in teaching parallel programming in secondary schools is rare, though a few authors offer their experiences: Y. Ben-David Kolikant [21], Torbert et al, [26]. Experience in teaching parallel programming to primary and secondary school students at IT camps and workshops has been described by Chesebrough and Turner [48], Gregg et al [49], Feldhausen [22].

### 3.1 Teaching programming in science-mathematics high schools in Croatia

The Fifth High School in Zagreb (V. gimnazija Zagreb) operates in line with the science-mathematics high school curriculum where, depending on the specialization, students have two or 3 h of informatics classes a week throughout the four years of schooling, that is totally 70 or 105 school hours per each year. The majority of informatics classes, especially in senior years, include programming. Some of the course content for programming offered in science-mathematics high schools, which prior to the adoption of the new curriculum were current [70] is as follows:

• Entering and printing data,
• Branching,
• Loops,
• Subprograms,
• Complex data types (array, strings, sets, files),
• Databases,
• Recursive programming,
• Dynamic data structures,
• Abstract data types (stack, queue, and tree),
• Computer graphics (drawing),
• Algorithm complexity.

Given that the curriculum over the last ten years has become outdated and replaced only upon adoption of the Course Curriculum – Informatics [71], teachers have had a certain amount of freedom in changing the curriculum. Hence, during the last 10 or so years, the following topics have been taught at the Fifth High School: object-oriented programming, programs with GUI, combinatorial algorithms (combination, permutation, partitions of a set), graph algorithms, cryptography, and experimenting with parallel programming, which is the subject of this research. Parallel programming is positioned as one of the last topics in (the final) Year Four of Secondary School.

### 3.2 Concepts, examples, and teaching methods

Niño [7] mentions fundamental decisions are required prior to teaching parallel programming:

• Selecting the program model,
• Programming language,
• Tools for testing and correcting errors in programs,
• Introductory problems and algorithms,
• How to introduce parallel programming.

Given that students have been programming in the Python programming language since the beginning of secondary school, and given that D. J. John [72] recommends that when teaching parallel programming, consideration should be given to the fact that students continue to work in the same or similar environment, selecting the programming language was relatively easy. Therefore, parallel programming was taught using the Python programming language. Various models for accessing parallel programming can be done in Python, some of which are MapReduce [28], CUDA [73], OpenMP and MPI [37], multithreading [37], and [27]. For the purpose of this research, the approach required selecting the threads and processes model for teaching. Some of the reasons for this are the following: a) it comes with the standard Python installation and there is no need to install it additionally, which can be demanding in some instances, b) it includes a very small number of simple commands, c) it is at the appropriate level in terms of student knowledge. In addition to the process model, the data parallelism model (all processes do the same task only on different datasets) was also chosen. The teaching was based on examples and software templates, which is in line with the results of the review article [74] and the research conducted by Capel et al, [75].

As is the case with teaching any other content, parallel programming requires introductory (motivational) examples to incite interest in students. As opposed to the majority of research, the decision was made in this study to facilitate motivation for parallel programming using two groups of examples:

• The necessity of threads for some network applications that students know well,
• Speedup of program execution.

Experience in using threads for network applications in classes is described by Goldwasser and Letscher [65]. It involves a series of applications aimed at showing the importance of using threads: a client application for obtaining the exact time from a server; creating a server that can accept a client, receive a message from a client and return the same message, a so-called echo server; chat server operating simultaneously with several clients; a chat client that should ‘wait’ for messages from the server and display them on the screen and ‘wait’ for a message which the client enters and sends to the server.

On the other hand, the importance of improved performance for motivational purposes in teaching parallel programming has been noted by many authors. Along those lines, Joiner [76] believe that demonstrating improved performance using parallel programming is important, showing it live on a specific example. It is also important that the examples used are well known to students [77] believes that there are two basic conditions to get students interested in parallel programming: demonstrate the obvious difference in performance and illustrate that difference using a proper program code. Similar attitudes are presented by [59] who believes students want to see specific examples and actual performance improvements. The opinion of [72] is that teaching parallel programming to students should demonstrate certain facts such as increased program performance using parallel programming, the different models of parallel programming, such as the two basic models - shared and distributed memory, obstacles significantly degrading parallel programming (synchronization and communication), and the need to practice the principles on computers.

When viewing aspects of parallel programming in terms of increasing program performance, Brown et al, [78] considers that the fundamental knowledge which students should master by learning parallel programming includes identifying possibilities of parallelism in a given problem and evaluating the applicability of various parallel strategies in solving problems, Gopalakrishnan et al, [79] listing the topics which should be covered when teaching parallel programming, such as discussions on parallel programming, illustrating increased performance, providing an overview of certain approaches to parallel programming (MPI, threads), and explaining the memory models. D. P. Bunde [63] believe that the fundamental concepts that should be taught in parallel programming are:

• Fork/join,
• Race condition,
• Parallel speedup,
• Load balance.

Based on the last three references, and specified as learning outcomes, the fundamental learning outcomes to be achieved in terms of teaching parallel programming are:

• Establish that parallel programming can significantly speed up program performance,
• Confirm why speedup of a program occurs when applying parallel programming,
• Explain the possibilities of parallelism using an actual problem,
• Explain the problem of race condition, problems that consequently occur because of the issue, and to describe the manner of resolving the issue
• Implement a parallel solution to the given problem using threads or processes.

#### 3.2.1 Introductory example – threads in network applications

The introductory example for teaching parallel programming was to create a network application using threads.

Network applications, in which the software is run on one or more servers, while the server can work with several clients simultaneously, are commonly used [80]. elaborates on the concept of mobile applications, which are partially run on the cellphone, and more complex parts of it are run on distributed, so-called edge servers. Nowadays, distributed database management systems (DDBMS) are also commonly used. Such systems include a single logical database physically distributed across different servers. Query optimization is especially demanding with such databases [29, 30]. Another example of such systems is the increasingly used so-called blockchain system, which entails public data, but does not rely on a single central server, and instead relies on P2P [31]. An example of using blockchain technology is provided by Tanwar et al, [32].

Network application, as an introductory example was chosen because students are familiar with it. Selected contents of this topic were implemented over the course of six class hours (about 6% of totally years hours), with 2 h held in an ordinary classroom environment, and two 2-h classes held in the informatics classroom where students worked on computers. A summary of the class hours:

1st *class hour (classroom)* – Students received explanations on fundamental concepts relating to computer networks: elements of a computer network, network protocols, IP addresses, DNS, network layers, and ports. After that, an overview of the Python module *socket* was given which contains functions and classes for working on a computer network. Finally, a client-server application was implemented where the server waits for the client to connect, after linking up the client to the server, the client waits for the user to enter a message, then the entered message is sent to the server, the server receives the message and all letters of the message are converted into large letters, and this received message is sent back to the client. The client displays the received message on the screen. Finally, the students were given homework requiring them to change the server part of the application so that the server continually receives users and establishes the described communication with them. The students did not have any major problems with the homework.

*2nd and 3rd hours (computer exercises)* – After a short review, the students were given the task to connect to the server (which was already activated on the teacher's computer), send their name, and finally read the message which the server sends and displays on the screen. After a certain number of students completed the task, they assisted students who did not independently solve it successfully. The majority completed the task autonomously. After finishing the task, another similar task followed: students had to create an application with GUI where the left side was to contain a select list and a label in the right section. The application was to connect to the server and receive a list of picture names from the server. The list contained names of pictures that were separated from one another by a vertical line (|), and the names of the pictures had to be entered into the existing select list (left side of the screen). After the user clicks on the name of the particular picture in the select list, the name of the picture is sent to the server and receives from the server a record of the picture as a series of bytes and presents the received data as a picture on the label. Given that it is a somewhat more demanding problem, it was divided into smaller tasks: creating the interface, retrieving the list of pictures from the server, sending the name of the pictures to the server, and retrieving the pictures, then displaying the pictures. After a more detailed analysis of each part, the students solved the problem autonomously, and after some time, some of the students wrote the solution on the projector-connected computer, explaining it to those who had not solved that part of the task. Accordingly, the speed at which the task was solved was adapted to the knowledge and abilities of the students. Those with better knowledge autonomously solved the task without participating in further analysis with the teacher, whereas the ones with poorer knowledge solved the task autonomously only after the detailed analysis was presented by the teacher, while students with the least knowledge and abilities copied the solution for the tasks from the projector. Most of these smaller tasks had already become familiar to the students, most often in a different form. An exception was downloading pictures from the server, where students were able either to autonomously research on how to do it or wait for a detailed analysis, and this part of the task also posed a problem in that not a single student was able to solve the task autonomously. It is important to note that for the second example, students did not know how to specifically implement the server part of the application.

4th *hour (classroom) –* The idea was to present the problem regarding when a server should operate with several clients, respond to their requests, and also accept new clients. For this purpose, the goal was to devise a client-server application where the client sends numbers to the server, and the server responds with messages whether the sent number is prime or complex. Given that, during a single connection to the server, the client can send larger quantities of numbers, and the server should be able to simultaneously maintain communication with several clients. This is the essence of the problem: how to get the server to simultaneously wait for messages from all clients and to respond to them as soon as receiving a message from a client. The situation would be a lot simpler if clients sent messages in proper sequence, but there are no rules established. Therefore, the general idea is to have ‘workers’ within the program, where each one executes a very simple task: waits for a message from a client and then responds to it. Here, the concept of the thread is encountered. The application was tested by getting students to devise the client application on a prepared laptop, and the server operation was tested with two instances of the client application on the laptop, and one instance on the computer on which the server was set up. The server displays a message on the screen for each request. The homework was to change the server part of the application so that the number of summed digits is received. The client part of the application had to be changed so that the program terminates operation when the entered number is less than 0. When a number less than 0 is sent, the task+ was to terminate the associated thread on the server.

5th *and* 6th *hours (computer exercises)* – the goal of these class hours was to set up a functional chat application, i.e., the client and server part of the application. The application was devised in the following steps:

• The server application which will continually accept clients, receive the name from the client and send a message to all received clients that a new person with the registered name has joined the chat,
• Create the client part of the application for this type of server application where messages from the server are printed in Python textual windows,
• Further develop the client application by adding a graphical user interface to it and display the messages received from the server in the designated text area,
• Further develop the server application whereby a message is anticipated for each client from the server, and each of the received messages from the client is forwarded to all other clients (as well as the name of the client sending the message),
• Further develop the client part of the application by adding a graphical user interface and a frame for text entry into which the user writes the message along with the button *Send* to send messages to the server. Accordingly, the client will also need two threads: receive messages from the server and display them in the designated test area and then send messages to the server.

Though it seems that this section has no relation to parallel programming, it does provide an understanding of threads and their uses.

#### 3.2.2 Processes as a ‘tool’ for program speedup

[63] provided a detailed description of experience gained in parallel programming using examples with prime numbers. Some of the basic steps are sequential counting of prime numbers up to 2 000 000, parallelism with errors (no join or lock), speedup with unequal segmentation, and storing prime numbers which are later used to verify whether the number is a prime number.

A similar motivational example for illustrating speedup and teaching fundamental concepts of parallel programming was also provided in this case. Prime numbers, though not very interesting to students, were chosen since they are very familiar with the various algorithms for generating numbers and counting them. On the other hand, they can be time demanding and these kinds of numbers illustrate the time differences in program execution. These classes are conducted over 6 class hours (about 6% of totally years hours), just like in network programming: 2 h in the classroom and two 2-h sessions as computer exercises. Instead of using threads in network programming, the focus is on processes (multiprocessing module), as it gives better speedup results.

1st *hour (classroom)* – Students received explanations for certain terms relating to processes, with some differences such as threads, functions for working with processes, initiating processes, and waiting for the completion of a process. Several specific examples are presented in this hour: 4 processes are initiated, each of them displays their ID and name, and waits for a certain (random) number of seconds, and finally displays the elapsed time. Processes are initiated manually, one by one, then using the list. Subsequently, an example is given using counting prime numbers up to 1 000 000. A function is written to return the number of prime numbers in the interval from *a* to *b*. Two types of programs are created:

• Serial program – calls the function with parameters equaling 2 and 1 000 000,
• Parallel program – creating two processes, where the first one counts prime numbers from 2 to 500 000, and the second one counts them from 500 001 to 1 000 000.

Program execution time was measured for both cases. The results indicated that the parallel program executed faster. The speedup was not at 50%, but a little over 30%. An analysis was done to uncover the reason for the mentioned speedup, and not a greater speedup (e.g., 50%). One of the reasons is that it may be due to unbalanced distribution of activities. Specifically, the second process has a much more demanding job. The numbers which that process verifies, whether they are prime or not, are much larger and consequently more time-intensive. Moreover, the results indicate that after we created the parallel version of the program, we do not know how many prime numbers there are between 2 and 1 000 000. This means using the processes, it is not possible to get the return value that the function executed within the process. In our discussion, we arrived at the idea of setting up a global variable to act as a ‘counter’ for all processes. Given that it involves processes, this variable in Python must be a special type, i.e., a value or array. After implementing this variable and executing the program, the results indicated that the parallel program ‘functions inefficiently’, meaning that each time a different solution is displayed which is always wrong. This reveals the problem associated with race condition and the need to lock variables. The homework required running the program on a computer at home, which was to identify the number of cores and test the program of counting prime numbers using two, four, eight, and sixteen processes.

*2nd and 3rd hours (computer exercises) –* During these 2 h, students solved a series of smaller parallel tasks, such as counting list elements, finding the smallest list element, counting all Armstrong numbers in a given interval, approximating values of the number π using the Monte Carlo method, and other tasks. As before, students solved the problems autonomously (on their own), analyzing the problem with the teacher, while students who successfully solved the tasks helped other students or wrote the solutions on a computer connected to a projector. In all the examples, the speedup was measured based on serial versions of the same program.

4th *hour (classroom)* – The class hour aimed to achieve further speedups. Hence, changes were made in the variable locking procedure (only once at the end of the function, instead of each time when a prime number occurs). Also, some other options were considered - the use of the Array object or parallel Queue. A quicker speedup was obtained if the jobs were distributed in a more balanced way. One way of doing this was to create a lot of smaller jobs and use the parallel object Pool. Each job in this case was a shorter interval (for instance, 100 numbers). This enabled the processes to get less demanding intervals (with smaller numbers), and subsequently, the more demanding processes were distributed in a more balanced manner.

5th *and* 6th *hours (computer exercises)* – The goal of these 2 h was to continue work on parallelizing some programs and analyzing the speedup, with the example processes mainly involving matrix operations.

As can be seen, students were, to a great extent, active participants during classes, especially evident during computer exercises where they mostly solved the tasks autonomously. This supports the opinion of A. Marowka [81] and A. L. Fisher [82] who believe that practical experience is exceptionally important when teaching parallel programming, meaning that students do the programming on their own, or the programming is done in groups so that students can actively participate in classes, thinking through the problems in order to arrive at a conclusion, based on previous experience and newly learned course content. This method nurtures a constructivist approach to learning parallel programming as advocated by Dolgopolovas et al, [83].

## 4 Conducted research

The goal of the research was to get answers to the following questions:

• *Is there a difference in attitude concerning course content on parallel programming and other compulsory class topics for students in science-mathematics high schools?*
• *Are students at science-mathematics high schools able to adopt some fundamental concepts of parallel programming and apply them in new situations?*

Responses to these two questions either accept or reject the following hypotheses:

• *The topic of parallel programming is equally interesting to students as other topics covered in the class curriculum for the informatics subject at science-mathematics high schools.*
• *Students consider parallel programming equally difficult as other topics covered in the class curriculum for the informatics subject at science-mathematics high schools.*
• *Students consider parallel programming equally useful for their future professional development as other topics covered in the class curriculum for the informatics subject at science-mathematics high schools.*
• *After teaching parallel programming, students will identify the concept of race condition in some other, new problems more successfully.*

The research was conducted in two phases.

• A *pilot study* was conducted with final year students at the Fifth High School in Zagreb, at the end of the 2013/2014 school year (28 students), 2014/2015 (22 students), 2015/2016 (47 students), and 2016/2017 (25 students).
• *Research in the 2019/2020 school year* was conducted to investigate two aspects:
  o Student attitudes towards class content on programming which students encountered during four years of schooling. In all, 40 students participated in this research, i.e., 19 students from the Fifth High School in Zagreb and 21 students from the Antun Gustav Matoš High School in Zabok (Gimnazija Antuna Gustava Matoša - Zabok).
  o The possibility of relating the concept of race condition to problems which they have not yet encountered. A total of 85 students participated in this research at the outset, and 59 in the final part of the research. The research was conducted at the Fifth High School in Zagreb (62 students in the initial testing and 22 students at the final testing), the Antun Gustav Matoš High School in Zabok (24 students in the initial testing and 26 students at the final testing), and the Second High School (II. gimnazija Zagreb) in Zagreb (12 students in the initial testing and 11 students at the final testing).

### 4.1 Student attitudes to class content on programming encountered during four years of schooling

The aim of this research was to identify student attitudes on the position of parallel programming in terms of overall education, specifically in terms of the *level of interest*, *level of difficulty,* and *usefulness for future professional development* using the 5-point Likert scale. These items were selected to be in accordance with expectancy theory [84], and are valid for objectives and evaluation of the teaching method and content presented in this study. Expectancy theory posits that valence (interest in our study), instrumentality (difficulty in our study), and expectancy (in our study). Valence pertains to the affective orientation towards an outcome, instrumentality pertains to a perception that a certain level of performance is required for a particular outcome, and expectancy pertains to a belief that a particular act will result in a particular outcome. In the educational context, Expectancy theory provides a useful guideline for a teacher to establish an environment that is optimal for student engagement (e.g. [85]). No such research has been conducted so far, and our study aims to provide some initial findings on the appropriateness of teaching parallel programming to secondary school students viewed from the perspective of students.

#### 4.1.1 Sample and procedure

A convenience sample was used that consists of 162 students from the Fifth High School in Zagreb and the Antun Gustav Matoš High School in Zabok^1^, which participated in this research for over 5 school years (2013/14, 2014/15, 2015/16, 2016/17 and 2019/20). Participation was completely voluntary, and no reward was offered for participation. The students participating in the research were final year students and received their schooling in line with the syllabus for science-mathematics high schools (see Table 1).

**Table 1:** Structure of respondents.

School year	School	M	F	Other	Total
2013/14	Fifth High School, Zagreb	23	5	0	28
2014/15	Fifth High School, Zagreb	11	11	0	22
2015/16	Fifth High School, Zagreb	31	16	0	47
2016/17	Fifth High School, Zagreb	16	9	0	25
2019/20	Fifth High School, Zagreb	12	7	0	19
2019/20	A. G. Matoš High School, Zabok	11	9	1	21

The observed class topics were:

• *Structural commands* – branch statements (*if*) and loops (*for* and *while*),
• *Complex data types* – includes content on lists, strings, sets, dictionaries, and files,
• *Turtle graphics* – creating simple drawings and animations using statements from the *turtle* module,
• *Object-oriented programming –* fundamental concepts relating to object-oriented programming, series of simpler examples and forcing its use in future tasks,
• *Abstract data structures –* queue, stack, binary tree, implementation and use in solving tasks,
• *Graphs algorithms* – defining and manner of recording graphs on a computer and the standard graph algorithms: BFS, DFS, Minimal Spanning Trees (Prim and Kruskal), shortest paths between a vertex to all other vertices (Dijsktra), maximum flow (Ford-Fulkerson)
• *Cryptography* – traditional cryptographic algorithms (Cezar, Vigenère, transposition, Affine) and their implementation as well as an overview of modern algorithms: DES and RSA,
• *GUI programs* – creating functional programs with standard elements from a graphical user interface (*tkinter* module)
• *Parallel programming* – described in the previous chapter,
• *Databases* – creating a database structure and SQL queries for manipulating data. Linking the database with Python (sqlite3 module).

The topic *graph algorithms* were taken by students from the 2014/2015 generation. Therefore, graph algorithms were taken by a total of 140 students.

The pilot study (Fifth High School in Zagreb, school years: 2013/2014, 2014/2015, 2015/2016 and 2016/2017) was conducted in the form of a paper survey, given out in the last hour of informatics to Year 4 students, which was anonymous and voluntary.

Research in the school year of 2019/2020, due to the COVID-19 pandemic, was online (via MS Forms) and was anonymous and voluntary, conducted immediately prior to the end of classes for Year 4 students.

Importantly, all classes in parallel programming in all research steps were conducted in classrooms (not online).

Data analysis was conducted using R [86]. Statistical analysis observed the entire research based on several divisions:

The first division was based on time and the spatial component of the research:

a. *pilot study*: 2013/2014, 2014/2015, 2015/2016 and 2016/2017 school years (PS),
b. *research conducted in the 2019/2020 school year at the Fifth High School in Zagreb (VHS)*,
c. *research conducted in the 2019/2020 school year at the A. G. Matoš High School in Zabok (AGHS).*

The second division was based on gender: male, female and other. Given that only one respondent was in the category other, this category dropped when doing analyses based on gender.

The third division was based on the *type of faculty/higher education institution they want to enroll into:*

a. Faculties at which informatics/programming is one of the central topics: Faculty of Science (Mathematics, Physics), Faculty of Electrical Engineering and Computer Science, Mechanical Engineering and the like (CS),
b. All other faculties (OT).

The structure of respondents based on the three divisions is shown in Table 2.

**Table 2:** Structure of respondents per division.

Division	n	%
*Time and spatial component*		
PS	122	75
VHS	19	12
AGHS	21	13
*Gender*		
Male	104	64
Female	57	35
Other	1	1
*Type of faculty*		
CS	98	60
OT	64	40

#### 4.1.2 Evaluating the level of interest in the course content

Students evaluated their level of interest in the course content using the 5-point Likert scale (1, 2, 3, 4, and 5), where 1 is *I did not find it interesting at all* and 5 is *I found it very interesting*. The arithmetic mean (M) and standard deviation (SD) of student evaluations are given in Table 3:

**Table 3:** Descriptive statistics for the level of interest.

Course content	M	SD	N
Structural statements	3.90	1.11	162
Complex data types	3.63	1.13	162
Turtle graphics	3.90	1.23	162
Object-oriented programming	3.81	1.16	162
Abstract data structures	3.37	1.21	162
Graph algorithms	3.21	1.23	140
Cryptography	4.00	1.13	162
GUI programming	4.17	1.10	161
Parallel programming	3.61	1.22	160
Databases	3.64	1.24	161

In order to test the first hypothesis, the Friedman test was selected to compare the level of interest across course content. Planned post hoc comparisons were done between parallel programming and other class topics by the Nemenyi test, as it controls for familywise error using the PMCMRplus package [87]. To check for potential group differences in the level of interest in parallel programming, it was compared across time and spatial component using the Kruskal-Wallis test, as well as across gender and type of faculty using the Mann-Whitney U-test. A threshold of p < 0.01 was selected for statistical significance. Non-parametric tests were selected as no assumptions for using parametric tests were fulfilled. As an effect size (ES) estimator, probability-based Vargha & Delaney's A was selected for the Mann-Whitney U-test, epsilon-squared was selected for the Kruskal-Wallis test, and Kendall's W was selected for the Friedman test. The first two effect size estimators were calculated using R package *rcompanion* [89], while the latter was calculated by hand. The default treatment of missing data by R was left for the Mann-Whitney U-test and Kruskal-Wallis test, while listwise data deletion was used for the Friedman test, as R requires complete data to apply it, and it was calculated on n = 134 students. The same selection of tests was used for the following two hypotheses. The associated statistic, p-value, and ES are shown in Table 4.

**Table 4:** Evaluation of interest in class content.

Comparison	*Statistic*	*p*	*ES*
Course content	109.83	0.000	0.091
Time and spatial component	1.204	0.548	0.008
Gender	3108.5	0.354	0.524
Type of faculty	1857.5	0.000	0.299

As shown in Table 4, no significant differences were found in the level of interest in parallel programming across time and spatial component and gender. However, a significant difference was found for the type of faculty students intend to enroll in (Mann-Whitney U = 1857.5, p < 0.01) with moderate to large ES (A = 0.30), where the students who intend to enroll into CS-related faculty consider parallel programming more interesting than other students. Also, a significant difference was found for level of interest in class content (Friedman chi-squared (9) = 109.83, p < 0.01) with small ES (W = 0.09). Post hoc comparisons showed that only GUI programming is evaluated as more interesting than parallel programming (cf. Table 3.). Therefore, the first hypothesis is partially confirmed.

#### 4.1.3 Evaluating the level of difficulty of the course content

Students evaluated the level of difficulty for the course content on a scale of 1–5, where 1 is *course content is not difficult at all* and 5 means *course content is very difficult.* The arithmetic mean (M) and standard deviation (SD) of student evaluations are given in Table 5:

**Table 5:** Descriptive statistics for the difficulty of class content.

Course content	M	SD	N
Structural statements	1.79	1.08	162
Complex data types	2.09	1.19	162
Turtle graphics	1.58	1.08	162
Object-oriented programming	2.48	1.22	162
Abstract data structures	2.51	1.16	162
Graph algorithms	2.62	1.14	140
Cryptography	2.18	1.14	162
GUI programming	2.23	1.20	162
Parallel programming	2.89	1.22	161
Databases	2.40	1.29	162

Comparing the evaluation of the difficulty of parallel programming with respect to other course content, and potential group differences in difficulty of class content across time and spatial, component, gender, and type of faculty, are given in Table 6.

**Table 6:** Evaluation of difficulty of class content.

Comparison	*Statistic*	*p*	*ES*
Course content	241.76	0.000	0.200
Time and spatial component	4.993	0.082	0.031
Gender	2729.5	0.503	0.460
Type of faculty	3539.5	0.082	0.570

As shown in Table 6, no significant differences were found in the evaluated difficulty of class content in parallel programming across time and spatial component, gender, and type of faculty. However, a significant difference was found for level of interest in class content (Friedman chi-squared (9) = 241.76, p < 0.01) with small ES (W = 0.20). Post hoc comparisons showed that most of the comparisons are significant, and that parallel programming is evaluated as more difficult than every other course topic (cf. Table 5). Therefore, the second hypothesis is not confirmed.

#### 4.1.4 Evaluation of the usefulness of class content for future professional development

Students also evaluated the usefulness of course content on a scale of 1–5, where 1 means that the course content *will not be of any use to future professional development*, and 5 means that the course content *will be of great use for future professional development.* The overall results in evaluating the complexity and the arithmetic mean are given in Table 6.

Comparing the evaluation of the usefulness of parallel programming with respect to other course content, and potential group differences in the difficulty of class content across time and spatial component, gender, and type of faculty, are given in Table 7.

**Table 7:** Evaluation of usefulness of parallel programming and other class topics.

Comparison	*Statistic*	*p*	*ES*
Course content	258.87	0.000	0.215
Time and spatial component	1.899	0.387	0.012
Gender	3317	0.127	0.560
Type of faculty	747	0.000	0.120

As shown in Table 7, no significant differences were found in evaluated usefulness of class content in parallel programming across gender, and type of faculty, but significant difference was found across the type of faculty the students want to enroll in (Mann-Whitney U = 747, p < 0.0001) with a moderate ES (epsilon-squared = 0.120). Furthermore, a significant difference was found for evaluation of the usefulness of course content (Friedman chi-squared (9) = 258.87, p < 0.01) with small ES (W = 0.215). (cf. Table 8). Post hoc comparisons showed that most of the comparisons are not significant, and that parallel programming is evaluated as equally useful as other course content except for turtle graphics, which is evaluated as less useful (cf. Table 5) This finding mostly supports the third hypothesis. Also, since the students who plan to enroll into faculties at which informatics/programming is one of the central topics consider it more useful than other students, this finding suggests that teaching of parallel programming might benefit from the higher focus on students who will likely require it for future professional development.

**Table 8:** Descriptive statistics for the evaluated usefulness of class content for further professional development.

Course content	M	SD	N
Structural statements	3.82	1.33	161
Complex data types	3.75	1.35	160
Turtle graphics	2.34	1.14	160
Object-oriented programming	3.58	1.38	161
Abstract data structures	3.16	1.33	161
Graph algorithms	3.09	1.41	139
Cryptography	3.07	1.40	161
GUI programming	3.41	1.40	161
Parallel programming	3.60	1.43	160
Databases	3.84	1.35	161

### 4.2 The possibility of transferring the concept of race condition to other problems

Lewandowski et al, [67] conducted a study on 66 students ranging from 18 to 20 years of age at 5 universities. The students were given the task of creating a system for concert ticket reservations. The tickets are sold via telephone. When a person wants to buy *n* tickets, they call a telephone number which is answered by an operator who sells *n* seats in a hall where the concert is to be held. Students were required to describe the problems they encountered in this manner of purchasing tickets and how to solve the problems. The goal was to find out whether students will notice that the same places in the hall can be sold by different operators via telephone. The results showed that 97% of students identified the problem of possibly selling a seat multiple times, with 71% of students providing a reasonable solution. In addition, the results were similar among students who had experience in parallel programming and those without such experience.

While student attitudes are certainly informative, to further evaluate the appropriateness of teaching parallel programming to secondary school students, a second study was conducted. As previous authors have underscored practical experience in learning parallel programming [68, 69], which is in line with the constructivist approach [70], the second study includes a practical assignment.

#### 4.2.1 Sample and procedure

Using the example from [57], in the 2019/2020 school year, similar research was carried out in the following Croatian schools: Second High School in Zagreb, Fifth High School in Zagreb, and the Antun Gustav Matoš High School in Zabok. The students in the Fifth High School and in A. G. Matoš High School in their final year of secondary school were taught according to the syllabus for science-mathematics high schools where informatics is a compulsory subject throughout the 4 years of secondary school education. The students in the Second Grammar School were taught according to the syllabus for general high schools where informatics is a compulsory subject only in the first year of secondary school, whereas it can be chosen as a facultative subject. Hence, the sample includes students ranging from the second year to the fourth year of secondary school. Participation was voluntary and no reward was offered for participation.

The goal of the research establishes to what extent students are familiar with the concept of race condition before and after studying parallel programming. Students were divided into two groups (A and B). The students were divided pseudo-randomly into equally-sized groups. They were also informed about which group they belonged to. The research was carried out in two phases:

• *Initial research/testing –* immediately prior to teaching parallel programming,
• *Final research/testing* – at the end of the school year (after teaching parallel programming).

The group of students in a particular phase of research solved one or more of the following tasks:

• The first task involves parallel programming and identifying the concept of race condition, with the task description as follows:

Two employees work on cinema ticket reservations over the phone. Users call them to book cinema tickets. The employee has his papers showing the layout of the cinema with seating locations. When a person calls to make a reservation or buy a ticket, the persons say which films they want to see and which seats they want to reserve.

Depending on the occupancy of these seats, the employee will make the reservation.

Describe the possible problems which will occur for making these reservations/purchases of cinema tickets. If you notice some problems, explain how you intend to solve them? How would you solve the problem more efficiently?

• The second task was a control task and in terms of this research, it was not important:

The owner of an apple orchard wants to plant one more area of apples. He has employed several workers for the task and they will dig holes in the soil to plant the apple trees. Devise a strategy so that the owner of the orchard can employ the optimal number of workers who will dig the hole in the shortest time possible. Cite an example of a hole-digging strategy which is not optimal.

Group A solved Task 1 in the initial research/test, while Group B received Task 2 in the initial research/test. After teaching parallel programming, that is, at the end of the school year, students solved the same tasks, only the tasks were switched in the groups so that Group A got Task 2 and Group B got Task 1.

A total of 85 students participated in the initial research, with 42 students in Group A and 43 students in Group B. The initial research was conducted in the classroom and on paper, it was anonymous and voluntary.

The structure of students is given in Table 9:

**Table 9:** Initial research – the structure of students by school and group.

School	A	B	Total
Fifth High School, Zagreb	24	25	49
A. G. Matoš High School, Zabok	13	11	24
Second High School, Zagreb	5	7	12
Total	42	43	85

In the final research, a total of 59 students participated, whose structure is given in Table 10 (see Tables 11 and 12).

**Table 10:** Final research – the structure of students by school and group.

School	A	B	Total
Fifth High School, Zagreb	9	13	22
A. G. Matoš High School, Zabok	10	16	26
Second High School, Zagreb	4	7	11
Total	23	36	59

**Table 11:** Results of the initial research by school and group.

School	Identified the race condition problem	Did not identify the race condition problem	Total
Fifth High School, Zagreb	19	5	24
A. G. Matoš High School, Zabok	10	3	13
Second High School, Zagreb	3	2	5
Total	32	10	42

**Table 12:** Results of the final research by school and group.

School	Identified the race condition problem	Did not identify the race condition problem	Total
Fifth High School, Zagreb	13	0	13
A. G. Matoš High School, Zabok	15	1	16
Second High School, Zagreb	7	0	7
Total	35	1	36

In the initial research, a total of 33 students (79%) noticed that a possible problem occurred if two persons wanted to reserve the same seat, while persons working on the telephones do not have synchronized papers to work with. All students gave the correct approach to solving the problem. In general, the solution required creating an application that would avoid those problems. Some other solutions were that each worker reserves seats for a section of the cinema and that there be constant communication among workers. Using the Fisher exact test with two-sided hypothesis testing, it was established that in the initial research, there was no statistically significant difference in identifying a race condition in terms of the school which the students attend (p = 0.674).

The final research was conducted online due to the COVID-19 pandemic but was also anonymous and voluntary. In the final research, a total of 35 respondents (97%) recognized the race condition problem where a seat may be reserved twice, and they all described a manner of solving the problem mostly identical to solutions in the initial research. Another interesting solution was that each person on the telephone reserve tickets for different films. The Fisher exact test with two-sided hypothesis testing did not show a statistically significant difference in identifying the race condition, in terms of the school which the students attended (p = 1.00).

A comparison of the initial and final testing using Fisher exact test with two-sided hypothesis testing shows that there exists a statistically significant difference among the results in identifying the race condition (p = 0.009) and it becomes evident that a greater percentage of students in the final research/test identified the race condition. Therefore, the fourth hypothesis is confirmed.

## 5 Discussion

Gauging the level of students’ interest in different course topics has shown that there is no significant difference whether observing students in the time (school year) or location (Fifth High School Zagreb and A. G. Matoš High School) triangulation. Also, there is no statistically significant difference in the level of interest in course content in terms of student gender. A statistically significant difference occurs for most course content in terms of the field students want to get into in the future (faculties where computer science or programming is a central topic (CS) and other faculties (OT)), while comparing the arithmetic mean shows that students aiming for a CS field consider most of the course content to be more interesting than in the case of students who intend to enroll into another field (OT). Accordingly, we can conclude that parallel programming is equally interesting to students who participated in preliminary testing, students from the Fifth High School in Zagreb for the 2019/2020 school year, and students from the A. G. Matoš High School in Zabok for the 2019/2020 school year (Kruskal-Wallis chi-squared = 1.203, p = 0.548) in terms of gender (Mann-Whitney U = 3108.5, p = 0.354). On the other hand, given the field into which they intend to enter, parallel programming shows a statistically significant difference between students who intend to enter the CS field and those who plan to get involved in other fields (Mann-Whitney U = 1857.5, p = 2.821e-05) with moderate to large ES (A = 0.30), where students who want to enter the CS field, find parallel programming more interesting than other students. When referring to the level of interest in parallel programming and other topics as shown in Table 4, it becomes evident that this course content is as interesting as most of the other topics, which mostly confirms the first hypothesis.

The results from evaluating the degree of difficulty of parallel programming do not differ for any group of students, with students considering parallel programming to be more difficult than most other topics. Therefore, the second hypothesis is rejected.

When referring to the evaluation of the usefulness of the course content for future schooling or work with respect to parallel programming (but also most other topics), again there are no statistically significant differences for the time and location component (Mann-Whitney U = 1.899, p-value = 0.387) and gender (Mann-Whitney U = 3317, p = 0.127). A statistically significant difference was observed in terms of the field in which students plan to continue their education (Mann-Whitney U = 749, p = 2.2e-16). This finding suggests that if parallel programming is to be taught to secondary school students, additional attention should be given to those students who consider it more useful for their future development. Given the evaluation of usefulness, there exists a statistically significant difference if viewing all the course topics. In comparing parallel programming and other topics, the obtained results indicate that parallel programming is evaluated as equally useful as other course content except for turtle graphics. Therefore, the third hypothesis is mostly confirmed.

In terms of the ability to identify the concept of race condition before and after teaching parallel programming, there was no statistically significant difference in the results when students are observed in terms of their school at both initial (Fisher exact test, p = 0.674) and final testing (Fisher exact test, p = 1).

However, if referring to differences at the initial and final testing (before and after teaching parallel programming), students in the final testing significantly more successfully identified the problem of the race condition than in the initial testing (Fisher exact test, p < 0.01). Therefore, the fourth hypothesis is confirmed.

Our findings are in line with some previous research. For instance, Saraswat and Bruce [20] discovered that students can understand various synchronization problems. D. Kotz [57] found that beginner computer science students demonstrate common sense knowledge about important computer science topics and demonstrate intuition about parallel processes. Consequently, these findings indicate secondary school students can be easily taught advanced computer science topics.

Since the students are able to master the parallel programming assignments and consider them as interesting, and as useful for their future development as most other topics, but more difficult than most other topics, it appears that students would benefit from application of both expectancy theory and constructivist approach. The former can probably be more useful to facilitate student motivation, especially for the students who plan to enroll in a CS-related faculty, while the latter can probably be useful for practical assignments.

### 5.1 Open issues

Despite providing some novel insights as this research area is relatively unexplored, there are some notable limitations in the conducted research. First, the convenience sample, which is also relatively small, was used. Our research should be cross-validated with a probabilistic sampling and previously planned statistical power to achieve a more satisfactory sample size. Second, the items used to evaluate the teaching subject are not validated psychometric instruments. Therefore, future research should include a multi-item validated instrument (e.g. [88]) to enable a more thorough outcome evaluation. Finally, the use of nonparametric statistical techniques somewhat threatens the statistical conclusion validity.

In this paper, a single approach to parallel programming using Python and threads was described. In future research, some other concepts, such as MapReduce, could be investigated to see if secondary school students are able to master parallel programming concepts using a different approach. Furthermore, it might be interesting to investigate if the elementary school students can master parallel programming concepts, in line with [22]. Parallel programming concepts could be introduced to students using some graphical program (e.g. Scratch) or using robots. Another potential direction of future research is the comparison of parallel programming by using the examples in this study and some other approach to teaching parallel programming (e.g. parallel design).

## 6 Conclusion

Parallel programming has become the standard. This is due to an increasing number of devices having two or more processors, or two or more processor cores. A large number of universities have noticed the importance of parallel programming and included it into existing programs in the earlier years of study programs or have created special parallel programming courses for earlier years of study programs. There have been several endeavors in teaching parallel programming at various camps for students and in secondary schools. The question remained whether students below the undergraduate level would indeed understand the concept of parallel programming.

The results suggest that parallel programming is no less interesting to students compared to most other topics covered in the curriculum for the informatics subject in science-mathematics high schools. Also, students rank parallel programming as equally useful as most other topics. In terms of difficulty, it is evaluated as more difficult than other topics. Still, the results suggest students are able to identify and apply some of its fundamental concepts to other problems.

Based on the results, we can conclude that in addition to the described manner of teaching at science-mathematics high schools, it appears parallel programming can be taught in high schools, perhaps as a facultative subject.

## Declarations

### Author contribution statement

Predrag Brođanac: Conceived and designed the experiments; Performed the experiments; Contributed reagents, materials, analysis tools or data; Wrote the paper.

Josip Novak: Analyzed and interpreted the data; Wrote the paper.

Ivica Boljat: Conceived and designed the experiments.

### Funding statement

This research did not receive any specific grant from funding agencies in the public, commercial, or not-for-profit sectors.

### Data availability statement

Data will be made available on request.

### Declaration of interests statement

The authors declare no conflict of interest.

### Additional information

No additional information is available for this paper.

## Footnotes

1 More schools were supposed to participate in the study, but due to the COVID-19 pandemic, they were not able to conduct the classes live as described.
